# Decreased Expression of CXCR4 Chemokine Receptor in Bone Marrow after Chemotherapy in Patients with Non-Hodgkin Lymphomas Is a Good Prognostic Factor

**DOI:** 10.1371/journal.pone.0098194

**Published:** 2014-05-23

**Authors:** Grzegorz Mazur, Aleksandra Butrym, Ilona Kryczek, Dorota Dlubek, Emilia Jaskula, Andrzej Lange, Kazimierz Kuliczkowski, Michal Jelen

**Affiliations:** 1 Dept. of Internal and Occupational Diseases and Hypertension, Wroclaw Medical University, Wroclaw, Poland; 2 Dept. of Haematology, Blood Neoplasms and Bone Marrow Transplantation, Wroclaw Medical University, Wroclaw, Poland; 3 Department of Physiology, Wroclaw Medical University, Wroclaw, Poland; 4 Department of Clinical Immunology, L. Hirszfeld Institute of Immunology and Experimental Therapy, Polish Academy of Sciences, Wroclaw, Poland; 5 Division of Pathomorphology and Oncological Cytology, Wroclaw Medical University, Wroclaw, Poland; Mayo Clinic, United States of America

## Abstract

**Background:**

CXCR4 chemokine receptor is constitutively expressed on normal and malignant B lymphocytes derived from patients with B-cell lymphoproliferative disorders and has a significant role in cell migration to lymph nodes and bone marrow. Non-Hodgkin's lymphomas (NHL) constitute a heterogeneous group of lymphoproliferative diseases, which can localize not only to lymph nodes, but also can migrate to peripheral blood and metastase to other organs, including bone marrow.

**Aim:**

The purpose of this study was to determine CXCR4 gene expression in peripheral blood and bone marrow of NHL patients before and after treatment.

**Methods:**

Samples of lymphoma lymph nodes, peripheral blood and bone marrow aspirates of patients with B-cell NHL were taken at diagnosis and after chemotherapy. Gene expression was determined by the reverse transcription (RT)-polymerase chain reaction method. Expression was estimated from 0 AU (no amplificate signal) to 3 AU (maximal amplificate signal).

**Results:**

No significant difference in the level of CXCR4 expression was found in reactive lymph nodes compared to lymphoma samples We observed high level of CXCR4 expression in most patients before treatment: in bone marrow: 3 AU-10 pts, 2 AU–8 pts, 1 AU–2 pts. In peripheral blood: 3 AU–14 pts, 2 AU–4 pts, 1 AU–1 pts, 0 AU–1 pts. After chemotherapy, significant decrease in CXCR4 expression was observed. Bone marrow: 3 AU–5 pts, 2 AU–7 pts, 1 AU–5 pts, 0 AU–3 pts (p = 0.03). Peripheral blood: 3 AU–2 pts, 2 AU–6 pts, 1 AU–10 pts, 0 AU–2 pts (p = 0.0002). There was a good response to treatment in patients with significant decrease of CXCR4 expression in the bone marrow after treatment with 10-fold lower risk of death (p = 0.03).

**Conclusions:**

Decrease in CXCR4 expression in the bone marrow of NHL patients after chemotherapy may be a good prognostic factor.

## Introduction

Chemokines and their receptors have shown to be involved in cancer progression. CXCR4 chemokine receptor is widely expressed in normal tissues and plays an important role in development, mobilisation of haematopoietic stem cells, and trafficking of lymphocytes [1]. CXCR4 is constitutively expressed on normal and malignant B lymphocytes derived from patients with B-cell lymphoproliferative disorders and has significant role in cell migration to lymph nodes and bone marrow [2]. CXCR4 and its ligand CXCL12 play an important role in promotion of tumor growth in Ewing sarcoma [3] and mediate metastasis in ovarian, prostate and breast cancer [4]–[6]. Müller et al. showed lack of CXCR4 expression in normal breast tissue, whereas the same tumor-changed tissue was characterized by high expression of this receptor [7]. Kato et al. in his study observed a correlation between the expression of CXCR4 and the extent of metastasis of breast cancer to lymph nodes [6]. The role of CXCR4/CXCL12 axis has been proved also in hematopoietic neoplasms, such as acute lymphoblastic leukemia (ALL) [8], [9], acute myeloid leukemia (AML) [10], chronic lymphocytic leukemia (CLL), multiple myeloma (MM) and Waldenström Macroglobulinemia (WM) [11].

Non-Hodgkin's lymphomas (NHL) constitute a heterogeneous group of lymphoproliferative diseases, with different presenting features, clinical course and response to treatment. In Western countries majority of NHL's are B-cell origin. Lymphoma cells can localize not only to lymph nodes, but also can migrate to peripheral blood and metastase to other organs, including bone marrow.

The purpose of this study was to determine CXCR4 gene expression in lymphoma infiltrated lymph nodes in comparison to reactive lymph nodes. Also expression of CXCR4 was measured in peripheral blood and bone marrow of non-Hodgkin's lymphoma patients before and after treatment.

## Materials and Methods

### Ethics statement

The study was approved by the institutional review board of Wroclaw Medical University. Written informed consent was obtained from the patients before obtaining samples for this study.

### Lymph nodes

Chemokine gene expression was evaluated in 26 lymphoma lymph nodes taken from newly diagnosed patients (12 women, 14 men, aged 26–81 years; median age 57) before treatment. As a control group, 25 samples of reactive lymph nodes (taken from 15 women and 10 men, aged 18–59; median age 32) were also evaluated for chemokine gene expression Table 1. Clinical staging of lymphomas was performed according to Ann-Arbour classification: there were 5 patients in II stage of disease, 10 patients in III stage, and 11 patients in IV stage of the disease. There were ten patients with lymphomatous bone marrow infiltration. The risk groups according to international prognostic index (IPI) were as follows: low risk (IPI 1) - seven patients, low-intermediate (IPI-2) in fifteen patients and high-intermediate risk (IPI-3) in four lymphoma patients.

**Table 1:** Patient characteristics.

Lymphoma type	No. of patients	Sex (F/M)	Median age (range) years
**B-cell lymphomas**	**26**	**12/14**	**57 (26–81)**
FL	7		
MCL	4		
SLL	4		
DLBCL	4		
MZL	3		
BL	2		
MM	1		
HCL	1		

FL — follicular lymphoma; MCL — mantle cell lymphoma;SLL — small lymphocytic lymphoma; DLBCL — diffuse large B-cell lymphoma; MZL — marginal zone lymphoma; BL — Burkitt lymphoma;MM — multiple myeloma; HCL — hairy cell leukemia;

All patients after diagnosis have been treated with immunochemotherapy: indolent lymphomas with R-CVP and aggressive lymphomas with R-CHOP regimen.

Samples of the studied lymphomas (26) and reactive lymph node tissues (25) were divided into two parts. One was fixed in 10% buffered formalin and then embedded in paraffin. Sections were stained with hematoxylin and eosin and evaluated histopathologically. The second parts of the lymph nodes, used for gene expression analysis, were snap frozen in liquid nitrogen and stored at −70°C.

### Isolation of mononuclear cells from peripheral blood and bone marrow

Peripheral blood and bone marrow were diluted twice using RPMI 1640 culture fluid or Hanks liquid and then overlaid on a single density gradient (density: 1.077 g/ml, Lymphoprep, Nycomed, Oslo, Norway). Cells were centrifuged at 400 g for 30 min at 20°C. Mononuclear cells, located in the intermediate layer between Lymphoprep and plasma, were collected with a pipette. The cell suspension was centrifuged twice (200 g, 10 min, 4°C) for the first time in RPMI 1640 culture liquid, the second time in PBS without calcium and magnesium ion-PBS (Ca^2+^ Mg^2+^). The density of the resulting suspension of mononuclear cells in the fluid were determined in a Turk liquid, in Burker chamber.

### Chemokine gene expression analysis

Total RNA was extracted from the frozen tissue samples using Trizol reagent (Invitrogen Corp., Carlsbad, CA, USA). The amount of isolated total RNA was estimated quantitatively by spectrophotometric measurement and qualitatively by electrophoretical view. First-strand cDNA was synthesized using First-Strand Synthesis System (Stratagene, La Jolla, CA, USA) according to the manufacturer's specifications. cDNA used for PCR was standardized toward to b-actin mRNA. Briefly, b-actin was initially amplified and quantified with serial dilutions of cDNA from each sample. CXCR4 was then amplified in each sample containing identical amounts of b-actin mRNA. Gene expression was estimated in arbitrary units (AU) using a 0 to 3 point AU scale. High expression was defined as an AU of 2 or 3. PCR was conducted using primer pairs for CXCR4 (sense GAC CGC TAC CTG GCC ATC, antisense GGC AGC CAA CAG GCG AAG A, 345 bp).

Amplification was performed in a MJ Research PTC-200 Peltier thermal cycler DNA engine (MJ Research Inc, Watertown, MA, USA). The PCR conditions were 35 cycles at 95°C for 30 seconds, and 72°C for 30 seconds, followed by final extension at 72°C for ten minutes. The size of PCR products was determined by agarose gel electrophoresis.

### Semi-quantitative analysis of agarose gel electrophoresis

Comparative semi-quantitative analysis of the degree of bands saturation was performed using densitometric method with a gel documentation system Gel-Doc (Bio-Rad) and computer program Quantity One (Bio-Rad) for 1-D analyzing. Assuming conventional units [AU - arbitrary units], depending on the band saturation, patients were divided into four groups with the expression of CXCR4 expressed in value from 0 to 3.

### Statistical analyses

Univariate analyses were performed using Fisher's exact test or chi-square test. To determine significant differences in the receptor expression intensity with different dependent variables logistic regression with backward elimination method and the maximum likelihood criterion were used. In order to determine the significance of differences in the level of receptor expression before and after treatment a non-parametric Wilcoxon test of two dependent samples was applied. The survival analysis was performed using Cox proportional hazards model and cumulative survival was analyzed via Kaplan–Meier plots. Probability values <0.05 were considered statistically significant and those between 0.05 and 0.1 as indicative of a trend. Statistical analysis was performed using Statistica for Windows (version 7.0) software (Sta-Soft Inc., Tulsa, OK, USA).

## Results

### CXCR4 gene expression in lymph nodes before treatment

#### Lymph nodes

**Figure 1:**
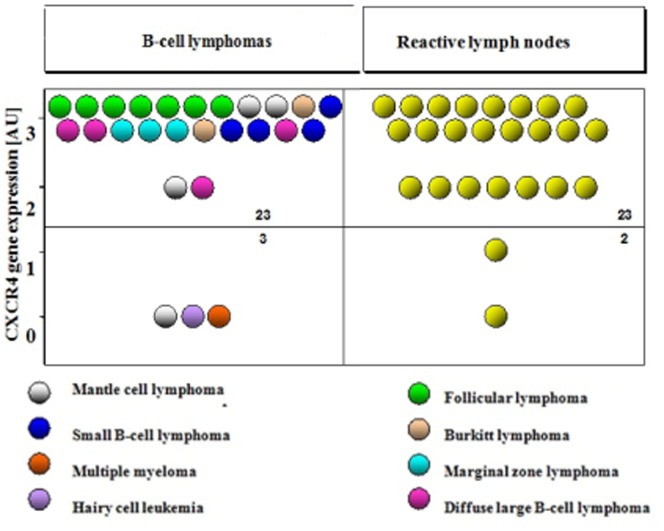
Expression of CXCR4 receptor gene in homogenates of lymphoma and reactive lymph nodes. Specific colors highlight histological types of lymphoma, (AU -arbitrary units)

In 46 out of 51 analyzed node homogenates (lymphoma and reactive lymph nodes) high CXCR4 expression was observed (Figure 1). No significant differences in the level of expression of this gene was found in reactive lymph nodes comparing to the lymphoma samples. Low CXCR4 gene expression was demonstrated only in 3 cases of lymphomatous lymph nodes. This frequency was comparable to that observed in the reactive nodes. Lymph nodes of patients with B-cell NHL with reduced (0 or 1 AU) expression of CXCR4 gene derived from patients with hairy cell leukemia, multiple myeloma and one patient with mantle cell lymphoma.

### CXCR4 gene expression in peripheral blood and bone marrow before and after treatment

Samples of peripheral blood and bone marrow aspirates of 20 patients (9 females, 11 males; aged 26–73, median age 57 years) with B-cell NHL (7 follicular lymphoma, 4 mantle cell lymphoma, 3 small lymphocytic lymphoma, 3 marginal zone lymphoma, 2 diffuse large B-cell lymphoma, 1 Burkitt lymphoma) were taken at diagnosis and after completed chemotherapy.

**Figure 2:**
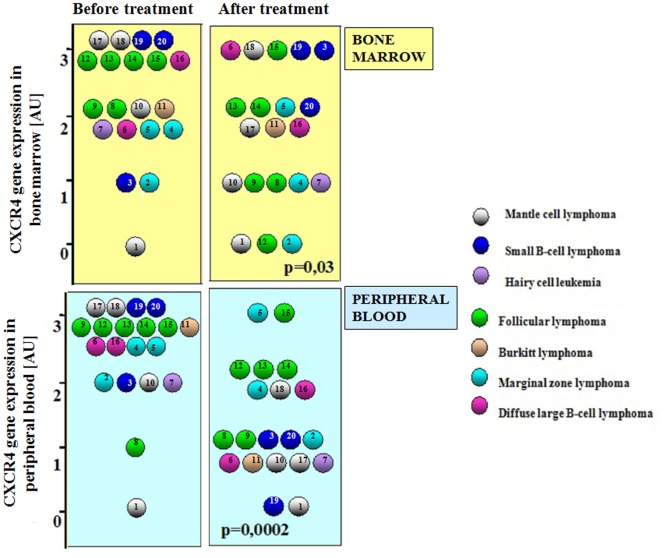
Expression of CXCR4 receptor gene in the bone marrow and blood of NHL patients. Expression before treatment and after treatment (arbitrary units - AU).

Most untreated patients presented with high CXCR4 expression levels in peripheral blood and bone marrow. It was comparable to the expression level observed in lymph nodes (Figure 2). The presence of lymphomatous bone marrow involvement was correlated with higher CXCR4 expression in the bone marrow before chemotherapy (p = 0.04).

It was observed that after effective treatment the strength of gene expression in both the bone marrow (p = 0.03) and peripheral blood (p = 0.0002) of patients with non-Hodgkin B-cell lymphomas was significantly reduced. Before treatment, CXCR4 gene expression at the level of 3 AU was observed in 9 of the bone marrow samples tested, and at 2 AU and less in 11 samples.

After treatment decreased expression of the gene was observed in 15 analyzed samples, which represent 75% of the cases. The largest decrease was observed in lymphomas of germinal centers. In patients with decreased CXCR4 expression after treatment in the bone marrow there was high rate of complete remissions, in contrast to those without decreased expression (Table 2).

**Table 2:** Response evaluation and re-staging of lymphoma patients after therapy in relation to bone marrow CXCR4 expression changes.

	Patients with decrease of CXCR4 expression in the bone marrow (n = 15)	Patients with increase or no changes in CXCR4 expression in the bone marrow (n = 5)
**Response evaluation after therapy**		
Complete remission	11	0
Partial response	4	1
Stable disease	0	2
Progressive disease	0	2
**Re-staging after treatment according to Ann-Arbour**		
No evidence of disease	11	0
I	4	0
II	0	0
III	0	1
IV	0	4

**Figure 3:**
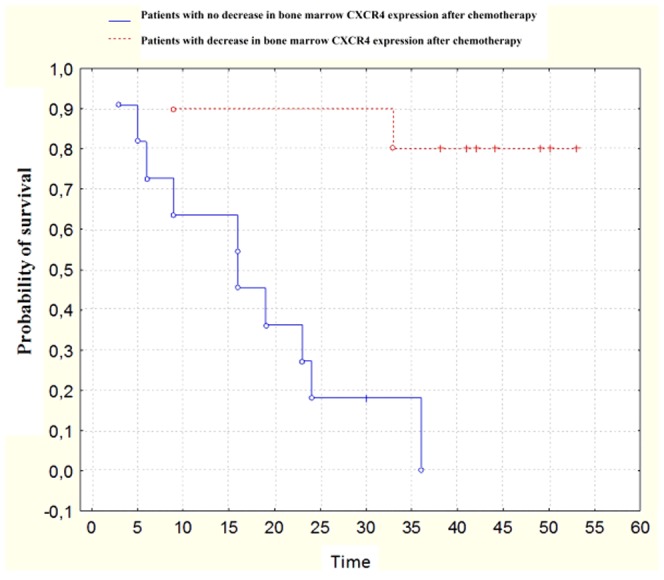
The cumulative proportion of patients' survival. Kaplan-Meier survival of B-NHL patients with the following levels of CXCR4 expression in bone marrow after treatment: there was no decrease − blue line, there was a decrease of CXCR4 expression − red dashed.

The most important finding was the influence of decreased CXCR4 gene expression in bone marrow after treatment on patients' response to therapy and survival. Before treatment, the level of CXCR4 gene expression in the bone marrow was high. Pre-treatment CXCR4 expression level was not correlated with patients' outcome. In some patients after treatment the expression of this receptor in the bone marrow was significantly lower. The group of these patients responded very well to therapy and had significantly better prognosis of survival. Reduced expression of CXCR4 in the bone marrow after treatment resulted in about a 10-fold decrease in the risk of death (p = 0.03). Figure 3.

## Discussion

CXCR4 receptor belongs to the family of G protein and it is constitutively expressed on normal and pathological B cells derived from patients with various B-cell neoplasms [4], [12], [13]. In the present study, we observed characteristic constellation of CXCR4 expression, which was increased in B cell lymphomas, as well as in reactive lymph nodes. Higher expression in reactive lymph nodes appears to be linked with an increase population of B-cells in inflammatory tissue.

CXCR4 and its ligand CXCL12 are important in the migration of cells to the lymph nodes in acute lymphoblastic leukemia and chronic lymphocytic leukemia [8], [9], [14], [15], they are also an important factor for migration of myeloma cells to the bone marrow [16]–[17]. In follicular lymphoma, CXCL12 enhances lymphoma cell movement [18]–[19]. Expression of CXCR4 and CXCL12 occurs commonly in normal tissues and plays a fundamental role in fetal development and the mobilization of hematopoietic cells and circulating naive lymphocytes [20]. Romboutus et al. demonstrated that CXCL12/CXCR4 may play an important role in the regulation of leukemic cells in acute myeloid leukemia and its increased expression is generally associated with a worse prognosis [21]. Other studies demonstrated, that downregulation of CXCR4 after CXCL12 binding resulted in strong inhibition of ALL cell line homing to the bone marrow [8].

In the present study, we showed expression of CXCR4 in almost all lymphoid tissues. Corcione et al demonstrated for the first time *in vitro* migration of malignant B cells in patients with follicular lymphoma in response to CXCL12 [12]. Other studies have shown that binding of CXCL12 to CXCR4 in mantle cell lymphoma promotes the migration of the cells and is associated with an invasive disease [4]. In our study there was no difference in the expression of CXCR4 between the group of aggressive and indolent B-cell lymphomas, which confirms wide dissemination of the CXCR4 receptor on B lineage cells.

Wong et al analyzed the expression of chemokine receptors in various lymphoproliferative disorders. They demonstrated a significantly lower level of CXCR4 expression in follicular lymphoma, in contrast to B-CLL, which was characterized by the high expression of that receptor [22]. The results obtained from studies of other groups illustrated higher expression of CXCR4 in B cell tumors with metastatic nodal involvement (CLL, MCL) than in tumors with limited location [5]. This observation would suggest that the CXCR4 receptor is responsible for nodal invasion. Another group found that CXCR4 expression was positive in all the analyzed cases of lymphomas, however, considerably higher in e MCL, B-CLL and HCL [14].

We showed that the average expression of CXCR4 in reactive lymph nodes is similar to that of the B-cell lymphoma nodes [23]. Helbig et al observed, that the nuclear factor NF-*κ*B acts as an inducer of the expression of CXCR4 on the cell surface [24]. On the other hand it is known, that inflammatory cytokines (such as interleukin-1, interleukin-8, tumor necrosis factor) are potent inducers of NF-*κ*B. This relationship may explain the relatively high expression of CXCR4 in reactive lymphoid tissue in our study.

We noticed that pretreatment bone marrow CXCR4 expression was correlated with bone marrow involvement. The same results had Deutsch et al, who also found this relationship [25]. But the most important results of our study showed, that lower expression of CXCR4 in the bone marrow after chemotherapy significantly influenced good response to therapy and longer survival. There are not many reports regarding expression of this receptor in bone marrow and peripheral blood of lymphoma patients. In the study of Mancuso et al. CXCR4 expression was analyzed in the bone marrow and peripheral blood of different non-Hodgkin lymphoma subtypes [26]. They noticed significant lower expression of CXCR4 in Waldenström Macroglobulinemia (WM) in comparison to other lymphomas. They did not show differences in CXCR4 in the bone marrow and peripheral blood between lymphoma types. Authors suggested the role of CXCR4 in hematogenous lymphoma dissemination. They observed lower CXCR4 expression in patients with diseases where circulating lymphoma B-cells are found less frequently (diffuse large B-cell lymphoma or WM) [11]. Higher expression was found in lymphomas with tendency to circulate in peripheral blood (chronic lymphocytic leukemia or follicular lymphoma) [24]. Deutsch et al. found CXCR4 expression in nodal marginal B-cell lymphomas and nodal diffuse large B-cell lymphomas, but not at extranodal manifestation sites [25]. Also in the present study we observed higher CXCR4 expression in follicular lymphomas, small B-cell lymphomas and mantle cell lymphomas, which have predisposition to spread into the blood.

The most significant finding of our study was the impact of decreased CXCR4 expression in the bone marrow after treatment on patients' survival. Patients with decreased expression had significantly longer survival compared to patients with higher expression.

Role of CXCR4 in metastatic process has been analyzed in many cancers [25], [27]–[28]. On the other side we know, that its expression occurs commonly in normal tissues and plays a fundamental role in lymphocyte circulation [20], [26], [29]. But we still do not know what is the role of microenvironment in supporting cancer progression. It is known, that CXCR4 expression can be enhanced by additional factors, such as hipoxia, wich up-regulates CXCR4 expression on cancer cells [30]. Basing on our results it is difficult to decide, if highly expressed CXCR4 reflects lymphoma tumor burden or mirrors the role of host immune cells. Once we showed, that in our lymphoma samples before therapy CXCR4 expression was high and it was comparable to CXCR4 expression in reactive lymph nodes, it can be determined with a large probability that this chemokine receptor is expressed by host microenvironment cells. No differ in the CXCR4 expression between normal lymphocytes and cancer cells shows, that the change in chemokine receptor expression should not reflect what is the proportion between normal lymphocytes and lymphoma cells, but rather be a reflection of microenvironmental regulation.

Protection from apoptosis and chemoresistance mediated by microenvironment and CXCR4/CXCL12 axis is well known in ALL or CLL, but not known yet in other NHL subtypes. In recent paper of Beider et al. again CXCR4 expression has been confirmed on lymphoma cells deriving from bone marrow biopsies [31]. Authors proved CXCR4 role in NHL progression and demonstrated, that using CXCR4-antagonist (BKT140) has potent anti-lymphoma activity, by induction of apoptosis. This observation would be indirectly in line with our results - we showed, that after successful treatment expression of CXCR4 decreased dramatically and it had potent impact on patients' survival. Beider et al. also showed role of CXCR4/CXCL12 axis in specific migration of lymphoma cells to the bone marrow and lymphoma cell protection from immunotherapy with monoclonal antibody against CD20 - rituximab [31]. These observations support our finding that decreased CXCR4 expression in the bone marrow after treatment increased survival compared to patients with higher expression. Decreased CXCR4 resulted in disrupt lymphoma and host bone marrow-derived stromal cells CXCR4/CXCL12 axis, what resulted in stroma-induced protection from immunotherapy infringement.

Taken together, a decrease in CXCR4 expression in the bone marrow of B-NHL patients after chemotherapy may be a good prognostic factor. These data require further evaluation on bigger and more homogenous group of lymphoma samples.

## References

[1] Balkwill F (2004) Cancer and the chemokine network. Nat Rev Cancer 4: 540–550. 15229479 10.1038/nrc1388

[2] Ratajczak MZ, Kim C, Janowska-Wieczorek A, Ratajczak J (2012) The expanding family of bone marrow homing factors for hematopoietic stem cells: stromal derived factor 1 is not the only player in the game. ScientificWorld Journal 10.1100/2012/758512 PMC3373139 22701372

[3] Berghuis D, Schilham MW, Santos SJ, Savola S, Knowles HJ, et al (2012) The CXCR4-CXCL12 axis in Ewing sarcoma: promotion of tumor growth rather than metastatic disease. Clin Sarcoma Res 2: 24 10.1186/2045-3329-2-24 23249693 PMC3549731

[4] Darash-Yahana M, Piskarsky E, Abramovitch R, Zeira E, Pal B, et al (2004) Role of high expression levels of CXCR4 in tumor growth, vascularization, and metastasis. FASEB J 18: 1240–1242. 15180966 10.1096/fj.03-0935fje

[5] Arya M, Patel HL, McGurk C, Tatoud R, Klocker H, et al (2004) The importance of the CXCL12-CXCR4 chemokine ligand-receptor interaction in prostate cancer metastasis. J Exp Ther Oncol 4: 291–303. 15844659

[6] Kato M, Kitayama J, Kazama S, Nagawa H (2003) Expression pattern of CXC chemokine receptor–4 is correlated with lymph node metastasis in human invasive ductal carcinoma. Breast Cancer Res 5: R144–R150. 12927045 10.1186/bcr627 PMC314431

[7] Müller A, Homey B, Soto H, Ge N, Catron D, et al (2001) Involvement of chemokine receptors in breast cancer metastasis. Nature 410: 50–56. 11242036 10.1038/35065016

[8] Juarez JG, Thien M, Dela Pena A, Baraz R, Bradstock KF, et al (2009) CXCR4 mediates the homing of B cell progenitor acute lymphoblastic leukaemia cells to the bone marrow via activation of p38MAPK. Br J Haematol 145: 491–499. 19344405 10.1111/j.1365-2141.2009.07648.x

[9] Tesfai Y, Ford J, Carter KW, Firth MJ, O'Leary RA, et al (2012) Interactions between acute lymphoblastic leukemia and bone marrow stromal cells influence response to therapy. Leuk Res 36: 299–306. 21889797 10.1016/j.leukres.2011.08.001

[10] Peled A, Tavor S (2013) Role of CXCR4 in the pathogenesis of acute myeloid leukemia. Theranostics 3: 34–39 10.7150/thno.5150 23382784 PMC3563079

[11] Ngo HT, Leleu X, Lee J, Jia X, Melhem M, et al (2008) SDF-1/CXCR4 and VLA-4 interaction regulates homing in Waldenstrom macroglobulinemia. Blood 112: 150–158 10.1182/blood-2007-12-129395 18448868 PMC2435685

[12] Corcione A, Arduino N, Ferretti E, Raffaghello L, Roncella S, et al (2004) CCL19 and CXCL12 trigger in vitro chemotaxis of human mantle cell lymphoma B cells. Clin Cancer Res 10: 964–971. 14871974 10.1158/1078-0432.ccr-1182-3

[13] Lopez-Giral S, Quintana NE, Cabrerizo M, Alfonso-Perez M, Sala-Valdez M, et al (2004) Chemokine receptors that mediate B cell homing to secondary lymphoid tissues are highly expressed in B cell chronic lymphocytic leukaemia and non-Hodgkin lymphomas with widespread nodular dissemination. J Leukocyte Biol 76: 1–10. 15155773 10.1189/jlb.1203652

[14] Ghobrial IM, Bone ND, Stenson MJ, Novak A, Hedin KE, et al (2004) Expression of the chemokine receptors CXCR4 and CCR7 and disease progression in B-cell chronic lymphocytic leukemia/small lymphocytic lymphoma. Mayo Clin Proc 79: 318–325. 15008605 10.4065/79.3.318

[15] Burger JA, Gribben JG (2014) The microenvironment in chronic lymphocytic leukemia (CLL) and other B cell malignancies: insight into disease biology and new targeted therapies. Semin Cancer Biol 24: 71–81. 24018164 10.1016/j.semcancer.2013.08.011

[16] Alsayed Y, Ngo H, Runnels J, Leleu X, Singha UK, et al (2007) Mechanisms of regulation of CXCR4/SDF-1 (CXCL12)-dependent migration and homing in multiple myeloma. Blood 109: 2708–2717. 17119115 10.1182/blood-2006-07-035857 PMC1852222

[17] Mazur G, Gębura K, Gieryng A, Butrym A, Wróbel T, et al (2013) The CXCL12-3'A allele plays a favourable role in patients with multiple myeloma. Cytokine 64: 422–426. 23711392 10.1016/j.cyto.2013.05.004

[18] Corcione A, Ottonello L, Tortolina G, Facchetti P, Airoldi I, et al (2000) Stromal cell-derived factor–1 as a chemoattractant for follicular center lymphoma B cells. J Natl Cancer Inst 92: 628–635. 10772680 10.1093/jnci/92.8.628

[19] Durig J, Schmucker U, Duhrsen U (2001) Differential expression of chemokine receptors in B cell malignancies. Leukemia 15: 752–756. 11368435 10.1038/sj.leu.2402107

[20] Moser B, Ebert L (2003) Lymphocyte traffic control by chemokines: follicular B heper T cells. Immunol Lett 85: 105–112. 12527215 10.1016/s0165-2478(02)00233-x

[21] Rombouts EJC, Pavic B, Löwenberg B, Ploemacher RE (2004) Relation between CXCR-4 expression, Flt3 mutations, and unfavourable prognosis of adult myeloid leukaemia. Blood 104: 550–557. 15054042 10.1182/blood-2004-02-0566

[22] Wong S, Fulcher DA (2004) Chemokine receptor expression in B-cell lymphoproliferative disorders. Leuk Lymphoma 45: 2491–2496. 15621766 10.1080/10428190410001723449

[23] Mazur G, Wróbel T, Hałoń A, Jeleń M, Butrym A, et al (2005) Chemokine receptors: CXCR3 (CD 183) and CXCR4 (CD184) expression in lymph nodes of lymphomas and lymphoid reactive tissue. Acta Haematol Pol 36: 113–119.

[24] Helbig G, Christopherson KW, Bhat-Nakshatri P, Kumar S, Kishimoto H, et al (2003) NF-*κ*B promotes breast cancer cell migration and metastasis by inducing the expression of the chemokine receptor CXCR4. J Biol Chem 278: 21631–21638. 12690099 10.1074/jbc.M300609200

[25] Deutsch AJ, Steinbauer E, Hofmann NA, Strunk D, Gerlza T, et al (2013) Chemokine receptors in gastric MALT lymphoma: loss of CXCR4 and upregulation of CXCR7 is associated with progression to diffuse large B-cell lymphoma. Mod Pathol 26: 182–194. 22936065 10.1038/modpathol.2012.134

[26] Mancuso P, Calleri A, Antoniotti P, Quarna J, Pruneri G, et al (2010) If it is in the marrow, is it also in the blood? An analysis of 1,000 paired samples from patients with B-cell non-Hodgkin lymphoma. BMC Cancer 10: 644 10.1186/1471-2407-10-644 21106070 PMC2995803

[27] Yasuoka H, Tsujimoto M, Yoshidome K, Nakahara M, Kodama R, et al (2008) Cytoplasmic CXCR4 expression in breast cancer: induction by nitric oxide and correlation with lymph node metastasis and poor prognosis. BMC Cancer 8: 340 10.1186/1471-2407-8-340 19025611 PMC2642845

[28] Caligaris-Cappio F (2003) Role of the microenvironment in chronic lymphocytic leukaemia. Br J Haematol 123: 380–388. 14616995 10.1046/j.1365-2141.2003.04679.x

[29] Busilloa JM, Benovica JL (2007) Regulation of CXCR4 Signaling. Biochim Biophys Acta 1768: 952–963. 17169327 10.1016/j.bbamem.2006.11.002 PMC1952230

[30] Guo M, Cai C, Zhao G, Qiu X, Zhao H, et al (2014) Hypoxia Promotes Migration and Induces CXCR4 Expression via HIF-1α Activation in Human Osteosarcoma. PLoS One 9: e90518 10.1371/journal.pone.0090518 24618817 PMC3949690

[31] Beider K, Ribakovsky E, Abraham M, Wald H, Weiss L, et al (2013) Targeting the CD20 and CXCR4 Pathways in Non Hodgkin Lymphoma with Rituximab and high affinity CXCR4 antagonist BKT140. Clin Cancer Res 19: 3495–507. 23637121 10.1158/1078-0432.CCR-12-3015

